# Fecal Carriage and Genetic Characterization of CTX-M-1/9/1-Producing *Escherichia coli* From Healthy Humans in Hangzhou, China

**DOI:** 10.3389/fmicb.2021.616687

**Published:** 2021-02-16

**Authors:** Jiawei Chen, Sheng Chen, Yin Jiang, Rong Zhang, Jiachang Cai

**Affiliations:** ^1^Clinical Microbiology Laboratory, The Second Affiliated Hospital of Zhejiang University School of Medicine, Zhejiang University, Hangzhou, China; ^2^Department of Infectious Diseases and Public Health, Jockey Club College of Veterinary Medicine and Life Sciences, City University of Hong Kong, Kowloon, Hong Kong; ^3^Clinical Laboratory, The First People’s Hospital of Fuyang Hangzhou, Hangzhou, China

**Keywords:** CTX-M-199, inhibitor resistance, hybrid, fecal colonization, plasmid replicon

## Abstract

CTX-M-199, a novel chimeric β-lactamase which mediated resistance to sulbactam and tazobactam, was recently identified in Hangzhou, China. This study investigated the prevalence of fecal carriage of bacteria producing CTX-M-199 and other CTX-M-1/9/1-type enzymes among healthy individuals and characterized the genetic features of *bla*_*CTX–M–*1/9/1_-bearing mobile elements. A total of 74 Enterobacterales strains carrying various *bla*_*CTX–M–*1/9/1_ genes, including *bla*_*CTX–M–*64_ (*n* = 40, carriage rate of 0.74%), *bla*_*CTX–M–*199_ (*n* = 23, 0.40%), *bla*_*CTX–M–*123_ (*n* = 5, 0.10%), novel *bla*_*CTX–M–*153_ (*n* = 5, 0.10%), and *bla*_*CTX–M–*132_ (*n* = 2, 0.04%), were isolated from 68 out of 5,000 (1.36%) fecal samples of healthy adults in Hangzhou City. Phylogenetic analysis based on whole-genome sequencing data showed that 72 *bla*_*CTX–M–*1/9/1_-bearing *Escherichia coli* isolates were clustered into four major clades, three of which included CTX-M-199 producers. Sixty out of 75 *bla*_*CTX–M*–1/9/1_ genes were located on plasmids belonging to four Inc types: IncI2, IncI1, IncFIB, and IncHI2. The *bla*_*CTX–M–*199_ genes were harbored by three of the four types of plasmids except for IncHI2. All these *bla*_*CTX–M–*1/9/1_ genes were carried on an IS*Ecp1*-mediated transposition unit. In conclusion, human fecal carriage of *bla*_*CTX–M–*1/9/1_ was low in healthy populations of China. The IS*Ecp1* was commonly associated with *bla*_*CTX–M–*1/9/1_ and may mediate its transmission on various mobile elements. Our findings provide insights into the dissemination and the development of further measures for the control of pathogens producing CTX-M-1/9/1-type enzymes.

## Introduction

β-Lactam antibiotics are currently the most widely used antibiotics in the treatment of infectious diseases (Bush and Bradford, 2016). The emergence and spread of extended-spectrum β-lactamases (ESBLs), which hydrolyze expanded-spectrum cephalosporins and monobactams, severely limit therapeutic options and pose a serious public health threat (Pitout and Laupland, 2008). β-Lactamase inhibitors, including clavulanate, sulbactam, tazobactam, and the new avibactam, with high affinity for β-lactamases, can greatly enhance the activity of their partner β-lactams against ESBL producers. Therefore, β-lactam/β-lactamase inhibitor (BLBLI) combinations can be considered for the treatment of non-severe infections (Gutierrez-Gutierrez and Rodriguez-Bano, 2019). Unfortunately, several TEM and SHV variants have been reported to confer resistance to BLBLIs since the 1990s (Drawz and Bonomo, 2010). These inhibitor-resistant variants have been found more frequently in Europe than in the USA and have never been reported in China (Canton et al., 2008; Manageiro et al., 2012). It is worrying that the inhibitor-resistant TEM enzymes were still prevalent in *Escherichia coli* isolates from both outpatients and hospitalized patients of a Spanish hospital (Rios et al., 2015). The first natural inhibitor-resistant CTX-M-type β-lactamase, CTX-M-190, has been found in *E. coli* from Shanghai, China, in 2017 (Shen et al., 2017). Soon after, we identified a chimeric CTX-M-64 variant carrying S130T substitution, CTX-M-199, which mediated resistance to sulbactam and tazobactam, in three *E. coli* strains from Hangzhou, China (Cai et al., 2017). Mass spectrometry and crystallography analysis revealed that the binding of the sulbactam to the active site rendered the formation of the inhibitor enzyme complex inefficient and resulted in inhibitor resistance in CTX-M-199 (Cheng et al., 2020). CTX-M-64, the first described chimeric CTX-M (a hybrid of CTX-M-14-like and CTX-M-15-like β-lactamases), was identified in a *Shigella sonnei* strain recovered from a Japanese tourist who had returned from China in 2009 (Nagano et al., 2009). Subsequently, several chimeric CTX-Ms, including CTX-M-64, CTX-M-123, CTX-M-132, and CTX-M-37, have been detected in *E. coli* from pets, food animals, and humans in China (Sun et al., 2010; He et al., 2013; Tian et al., 2014; Gu et al., 2015; Liu et al., 2015). CTX-M-64 or/and CTX-M-123 producers have also been reported in a domestic pet dog and wastewater in Japan (Kawamura et al., 2017; Tanaka et al., 2019), fecal samples from healthy children in Laos (Stoesser et al., 2015), and a patient in Germany (Pfeifer et al., 2018). Our previous study indicated that *bla*_*CTX–M–*199_ gene was located on the *mcr-1*-bearing IncI2 plasmid and harbored in *E. coli* isolated from stool samples (Cai et al., 2017). However, the prevalence of CTX-M-199 and other CTX-M-1/9/1 enzymes in fecal *E. coli* isolates from healthy populations and the range of plasmids carrying this gene are unclear. The study aims to determine the fecal carriage rates of CTX-M-1/9/1-producing Enterobacterales in the intestinal tract of healthy individuals and to depict potential mechanisms underlying the dissemination of the *bla*_*CTX–M–*1/9/1_ gene by investigating the genetic characterization of CTX-M-1/9/1 producers, *bla*_*CTX–M–*1/9/1_-bearing plasmids, and the genetic context of *bla*_*CTX–M–*1/9/1_ gene.

## Materials and Methods

### Isolation and Species Identification of Enterobacterales

A convenient sample of 5,000 stools collected from asymptomatic healthy adults (3,218 male and 1,782 female) receiving annual physical examinations in a hospital of Hangzhou, China, from July to September 2019 were investigated. In order to eliminate the effect of antibiotics or intestinal diseases on intestinal flora, participants showing any symptoms related to gastroenteritis or had been exposed to antimicrobial agents or to a hospital environment in the 3 months prior to sample collection were excluded from the study. Within 4 h of collection, 1 g of stool sample was inoculated into 5 ml of Luria–Bertani (Oxoid Ltd., Basingstoke, England) broth without shaking at 37°C for 24 h. Twenty microliters of the mixture was streaked onto a plate of China Blue agar (Hangzhou Binhe Microorganism Reagent Co., Ltd., Hangzhou, China) supplemented with 10 mg/L of cefotaxime and incubated at 37°C for 24 h for the selection of potential CTX-M producers. To avoid the omission of isolates carrying *bla*_*CTX–M–*1/9/1_ gene, suspected Enterobacterales with different colonial morphology were selected from each sample and sub-cultured on fresh selective medium. Thus, more than one Enterobacterales isolates were obtained from the same sample in some cases. Species identification of *bla*_*CTX–M–*1/9/1_-positive isolates was performed by MALDI-TOF MS (Bruker Daltonik GmbH, Bremen, Germany). This study was approved by the Ethics Committee of The Second Affiliated Hospital of Zhejiang University School of Medicine, and consent was given by the participants.

### Screening of *bla*_*CTX–M–*1/9/1_ Genes and Antimicrobial Susceptibility Testing

The *bla*_*CTX–M–*1/9/1_ hybrid genes, with the two ends matching the *bla*_*CTX–M–*1–like_ gene and the center matching the *bla*_*CTX–M–*9–like_ gene, were screened in Enterobacterales that grew on selective plates by PCR using primers of 5′-GCGTAGGT TCAGTGCGATC-3′ and 5′-AACCGTCACGCTGTTGTTAG-3′. These primers can amplify four known *bla*_*CTX–M–*1/9/1_ genes, including *bla*_*CTX–M–*64_, *bla*_*CTX–M–*199_, *bla*_*CTX–M–*123_, and *bla*_*CTX–M–*132_, but not *bla*_*CTX–M–*137_. The *bla*_*CTX–M–*1/9/1_-positive strains were subjected to PCR amplification and sequence analysis of the entire *bla*_*CTX–M–*1–group_ gene to determine the genotype as previously described (Yu et al., 2007). The minimal inhibitory concentrations (MICs) of 16 antimicrobial agents, including imipenem, meropenem, ertapenem, cefotaxime, cefotaxime/clavulanate, ceftazidime, ceftazidime/clavulanate, piperacillin/tazobactam, cefoperazone/sulbactam, ceftazidime/avibactam, cefmetazole, aztreonam, ciprofloxacin, gentamicin, tigecycline, and colistin, for *bla*_*CTX–M–*1/9/1_-positive isolates were determined using the broth microdilution method according to the CLSI guidelines (Clinical and Laboratory Standards Institute, 2018). Tigecycline susceptibility was interpreted using breakpoints for Enterobacterales as recommended by the US Food and Drug Administration^1^. The susceptibilities of the remaining 15 antimicrobial agents were interpreted according to CLSI recommendations (Clinical and Laboratory Standards Institute, 2020). The susceptibility breakpoints for cefotaxime, ceftazidime, and cefoperazone were applied for cefotaxime/clavulanate, ceftazidime/clavulanate, and cefoperazone/sulbactam. Chi-square test was used to compare the rates or proportions. A *p* < 0.05 was considered as statistically significant.

### Whole-Genome Sequencing and Genome Analysis

Genomic DNA extracted from *bla*_*CTX–M–*1/9/1_-positive isolates was sequenced using the MGISEQ-2000 platform (BGI Complete Genomics), and the reads were *de novo* assembled by SPAdes (v3.11.1) (Bankevich et al., 2012). The assembly scaffolds were subjected to a screening of *bla*_*CTX–M–*1/9/1_ genes. The contigs containing the target gene were searched in the NCBI database by the BLASTN program, and the putative gaps were filled by PCRs based on the available reference sequences in an attempt to obtain the complete plasmid sequences.

The obtained sequences were annotated with the RAST server, and plasmid types were identified by using PlasmidFinder 2.0, available at the Center for Genomic Epidemiology (CGE)^2^ (Carattoli et al., 2014; Overbeek et al., 2014). Sequence types and acquired antimicrobial resistance genes of CTX-M-1/9/1-producing isolates were identified at CGE using MLST and ResFinder 3.2, respectively (Larsen et al., 2012; Zankari et al., 2012). Comparisons of sequences from plasmids with the same Inc type were conducted using BRIG (v0.95) (Alikhan et al., 2011). Easyfig (v2.2.2) was used to visualize the linear alignment of the genetic structure of different *bla*_*CTX–M–*1/9/1_ genes (Sullivan et al., 2011). The Harvest suite was applied to run core-genome alignment and single-nucleotide polymorphism (SNP) calling, and a phylogenetic tree was constructed using Parsnp (Treangen et al., 2014). The generated phylogenetic tree was edited and visualized by iTOL (v3)^3^ (Letunic and Bork, 2016).

### The Transferability of *bla*_*CTX–M–*1/9/1_ Genes

The transferability of *bla*_*CTX–M–*1/9/1_ genes was also assessed by conjugation experiments which were carried out using filter mating method. Rifampin-resistant *E. coli* EC600 was used as the recipient strain. Transconjugants were selected on MacConkey agar plates containing 500 mg/L rifampin and 30 mg/L cefoperazone/sulbactam (2:1) or 20 mg/L aztreonam as appropriate.

### Experimental Evolution Assays

To assess the possibility of emergence of S130T substitution in CTX-M-64, *E. coli* transconjugants harboring *bla*_*CTX–M–*64_-carrying plasmids with different Inc types (including pM-64-1028, pM-64-826, pM-64-799, and pM-64-3814) were subjected to parallel experimental evolution assays in the presence of increasing concentrations of cefoperazone/sulbactam as previously described (Ripoll et al., 2011).

### Nucleotide Sequence Accession Numbers

Only one of the *bla*_*CTX–M–*1/9/1_-containing plasmids or chromosomal fragments sharing identical sequence or the same genetic structure surrounding *bla*_*CTX–M–*1/9/1_ has been deposited in GenBank. The accession numbers of 19 representative sequences are listed in Supplementary Table S3.

## Results

### Fecal Carriage of *bla*_*CTX–M–*1/9/1_-Positive Enterobacterales

Of the 5,000 stool samples, 1,755 isolates were obtained from 1,395 agar plates supplied with cefotaxime. Seventy-four Enterobacterales (including 72 *E. coli*, one *Klebsiella aerogenes*, and one *Enterobacter cloacae*) which were positive for the *bla*_*CTX–M–*1/9/1_ gene were isolated from 68 samples (Supplementary Table S1). The sequence chromatogram of the *bla*_*CTX–M–*1/9/1_ gene from *E. coli* 1028 showed double peaks at positions 334 (G/A) and 398 (G/C) of coding sequence (numbering from ATG), which suggested the presence of both *bla*_*CTX–M*–199_ and *bla*_*CTX–M*–64_ genes. To differentiate these two genes, a conjugation experiment was performed, and transconjugants carrying *bla*_*CTX–M*–199_ or *bla*_*CTX–M*–64_ gene were selected on different media containing cefoperazone/sulbactam (30 mg/L, 2:1) or aztreonam (20 mg/L), respectively. The analysis of 75 entire *bla*_*CTX–M*_ genes has identified 23 *bla*_*CTX–M*–199_, 40 *bla*_*CTX–M*–64_, five *bla*_*CTX–M*–123_, five *bla*_*CTX–M*–153_, and two *bla*_*CTX–M*–132_. The CTX-M-153 was a novel β-lactamase derived from CTX-M-123 by a single substitution of Ala for Pro at position 67 (A^67^P). Thus, the fecal carriage rate of *bla*_*CTX–M*–1/9/1_ was 1.36% (68/5,000), and those of *bla*_*CTX–M*–199_, *bla*_*CTX–M*–64_, *bla*_*CTX–M*–123_, *bla*_*CTX–M*–153_, and *bla*_*CTX–M*–132_ were 0.40% (20/5,000), 0.74% (37/5,000), 0.10% (5/5,000), 0.10% (5/5,000), and 0.04% (2/5,000), respectively. The fecal carriage rates of *bla*_*CTX–M*–1/9/1_ and *bla*_*CTX–M*–199_ among different age groups (21–30, 31–40, 41–50, 51–60, 61–70, and 71–0) varied from 0.80 to 1.99% and 0.16 to 0.80%, respectively (Supplementary Figure S1), and those for male and female were similar [1.37 vs. 1.35% for *bla*_*CTX–M–*1/9/1_ (*p* = 0.95) and 0.44 vs. 0.34% for *bla*_*CTX–M–*199_ (*p* = 0.60)].

Agar plates supplied with cefoperazone/sulbactam and aztreonam were used for the selection of transconjugants carrying the *bla*_*CTX–M*–199_ and other *bla*_*CTX–M*–1/9/1_ genes, respectively. Apart from *E. coli* 1028, *bla*_*CTX–M*–1/9/1_ genes from other 58 isolates were transferred to *E. coli* EC600. However, those from 15 *E. coli* isolates were not transferable (Supplementary Table S1).

### Antimicrobial Susceptibility Results

All 74 Enterobacterales were resistant to cefotaxime but were susceptible to tigecycline. Remarkably, resistance against carbapenems and colistin was observed in six (one CTX-M-199 producer, three CTX-M-64, and two CTX-M-153) and 22 (12 CTX-M-199, eight CTX-M-64, and two CTX-M-132) *E. coli* isolates, respectively. Regardless of their status of carbapenemase production, all *bla*_*CTX–M*–199_-positive *E. coli* isolates were resistant or intermediate to piperacillin/tazobactam and cefoperazone/sulbactam but were susceptible to carbapenems, cefotaxime/clavulanate, ceftazidime/clavulanate, ceftazidime/avibactam, and cefmetazole. These *E. coli* isolates were also susceptible or intermediate to aztreonam and ceftazidime except five isolates co-producing CTX-M-55. On the contrary, isolates carrying other *bla*_*CTX–M*–1/9/1_ genes were susceptible to piperacillin/tazobactam and cefoperazone/sulbactam but were resistant to aztreonam and ceftazidime. All isolates except for carbapenemase producers were susceptible to the compound of clavulanate or avibactam combined with their partner cephalosporins.

### Genomic and Phylogenetic Analysis of *bla*_*CTX–M–*1/9/1_-Positive Enterobacterales

**FIGURE 1:**
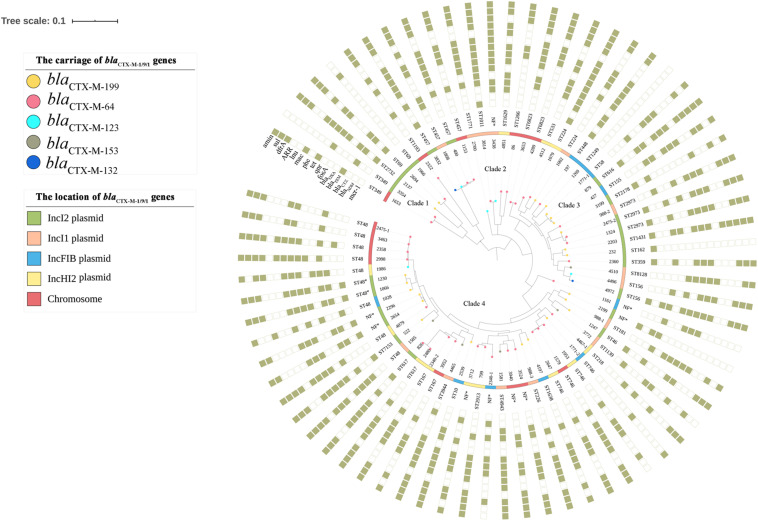
Phylogenetic tree of 72 *bla*_*CTX–M–*1/9/1_-positive *Escherichia coli* isolates, with their sequence types and profiles of acquired antimicrobial resistance genes. The genotype and the location of *bla*_*CTX–M–*1/9/1_ harbored by the isolate were illustrated. The rings consisting of small squares from the inside out indicated *mcr-1*, *bla*_*NDM*_, *bla*_*CTX–M*_ (except *bla*_*CTX–M–*1/9/1_), *bla*_*TEM*_, *bla*_*OXA*_, and genes conferring resistance to fosfomycin, quinolones, tetracyclines, phenicols, macrolides, lincosamides, rifampicin, trimethoprim, sulfonamides, and aminoglycosides, respectively. The filled square represented the presence of a resistance gene. NF, sequence type of the *E. coli* isolate was not found.

Seventy-four *bla*_*CTX–M*–1/9/1_-positive isolates and two *E. coli* transconjugants of *E. coli* 1028 which carried *bla*_*CTX–M*–199_ and *bla*_*CTX–M*–64_ genes, respectively, were subjected to whole-genome sequencing (WGS). The genome sizes of the tested isolates ranged from approximately 4.6 to 5.9 Mbp. The MLST analysis showed that 72 CTX-M-1/9/1-producing *E. coli* isolates belonged to a diverse range of sequence types, including 39 known STs and nine novel STs. Among 23 CTX-M-199 producers, a total of 21 STs were identified. ST48 was the most common type among these (*n* = 8), followed by ST457 (*n* = 4), ST746 (*n* = 4), and ST2973 (*n* = 3) (Supplementary Table S1). The phylogenetic analysis based on SNPs showed that 72 *E. coli* isolates were clustered into four major clades. The CTX-M-199 producers were grouped into three clades, and CTX-M-64 and CTX-M-123 producers can be found in each clade. Isolates belonging to the same ST were generally divided into one cluster (Figure 1).

The analysis of the antibiotic resistome of 74 *bla*_*CTX–M*–1/9/1_-positive Enterobacterales revealed a large range of resistance determinants, with 65 genes conferring resistance to 12 classes of antibiotics. The number of resistance genes harbored by each strain ranged from four to 23 (Supplementary Table S1). Apart from *bla*_*CTX*–M_, genes conferring resistance to aminoglycosides (*aac*, *aad*, *aph*, and *ant*), tetracyclines (*tet*), phenicols (*floR*), trimethoprim (*dfrA*), and sulfonamides (*sul*) were predominantly detected in the tested strains with positive rates of 87.8% (*n* = 65), 86.5% (*n* = 64), 82.4% (*n* = 61), 79.7% (*n* = 59), and 74.3% (*n* = 55), respectively. Fifty-seven plasmid-mediated quinolone resistance (PMQR) genes, including 27 *qnrS1*, 23 *oqxAB*, three *qnrS2*, three *aac(6′)-Ib-cr*, and one *qnrB6*, were identified (Supplementary Table S1). However, the carriage of PMQR genes was not entirely consistent with the ciprofloxacin resistance phenotypes. Chromosomal point mutations in quinolone resistance-determining region were examined using ResFinder 3.2 based on WGS data. Searching results showed the presence of mutations in GyrA [including S83L (*n* = 54), D87N (*n* = 37), and D87Y (*n* = 2)], ParC [including S80I (*n* = 46), A56T (*n* = 5), S80R (*n* = 1), E84G (*n* = 1), and E84A (*n* = 1)], or ParE [including S458A (*n* = 14), L416F (*n* = 2), and S458T (*n* = 1)] in isolates exhibiting high ciprofloxacin MICs (8 to >32 mg/L). Conversely, isolates carrying the PMQR gene alone were susceptible or intermediate to ciprofloxacin. The *bla*_*NDM*_ and *mcr-1* genes, which conferred resistance to two last-resort antibiotics against gram-negative bacteria, carbapenems and polymyxins, were detected in six and 22 *E. coli* isolates, respectively. These results were consistent with the MDR phenotypes observed among the tested strains. The *mcr-1* gene was detected more frequently in CTX-M-199 producers (12/23) than in non-CTX-M-199 producers (10/51), which resulted in a significantly higher (*p* < 0.05) resistance rate of colistin in the former (52.2 vs. 19.6%) (Supplementary Table S2).

### Genetic Characterization of *bla*_*CTX–M–*1/9/1_-Bearing Plasmids

Seventy-five DNA fragments harboring *bla*_*CTX–M*–1/9/1_ genes were obtained, among which 60 plasmids belonging to four Inc types, including 22 IncI2, 14 IncI1, 11 IncFIB, and 13 IncHI2, were identified in 58 isolates (strain 1028 carried two IncFIB plasmids encoding CTX-M-199 and CTX-M-64, respectively). The *bla*_*CTX–M–*199_ genes in this study were harbored by the first three types of plasmids. Fifteen *bla*_*CTX–M*–64_-containing DNA fragments, which ranged from 6 to 277 kb, were found to closely match known chromosomes, but not plasmid sequences, in the NCBI database by BLASTN, with coverage of >98% and identity of >99% (Supplementary Table S1).

**FIGURE 2:**
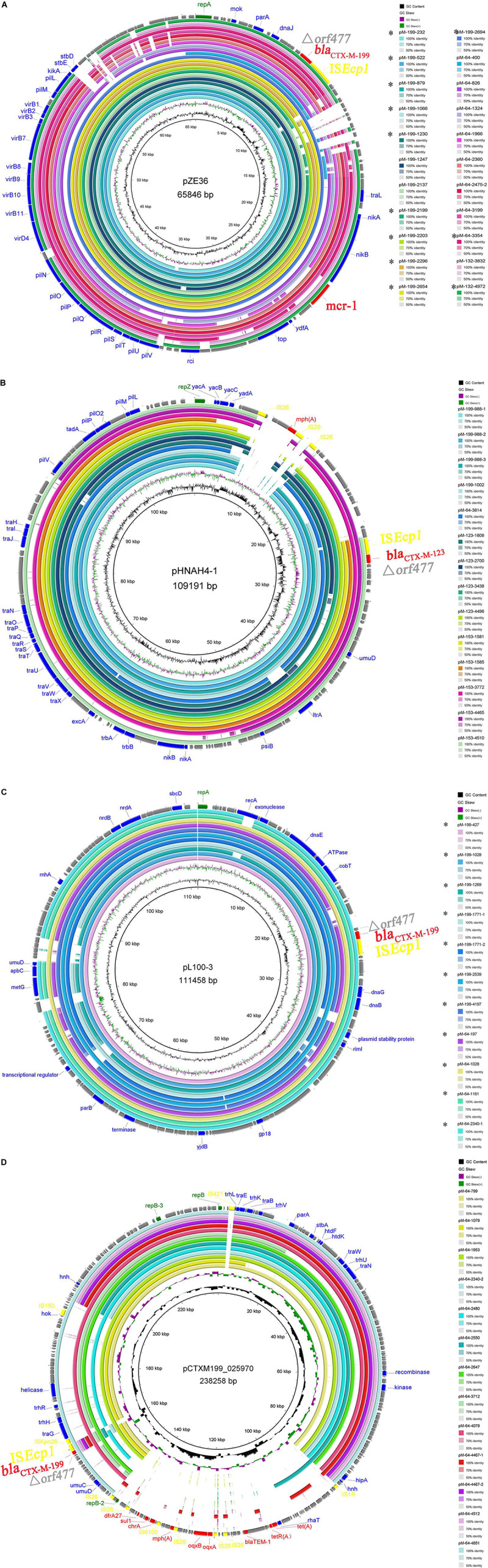
Comparisons of the assembled contigs of *bla*_*CTX–M–*1/9/1_-bearing plasmids with the same Inc type of IncI2 **(A)**, IncI1 **(B)**, IncFIB **(C)**, and IncHI2 **(D)**. Four reference plasmids with different Inc types were indicated in the middle of each circle. Circle 1 (innermost) displayed the scale in kilobase pairs. Circle 2 and circle 3 displayed the GC skew and GC content, respectively. The outermost circle displayed the partial annotation of the reference plasmid.

The sequence alignments showed that plasmids belonging to the same Inc type shared a high degree of sequence homology (Figure 2). IncI2 plasmid, in which the *bla*_*CTX–M–*199_ gene was firstly identified, was dominant among *bla*_*CTX–M–*1/9/1_-bearing plasmids, accounting for 12 *bla*_*CTX–M–*199_, eight *bla*_*CTX–M–*64_, and two *bla*_*CTX–M–*132_. Almost all these IncI2 plasmids carried the *mcr-1* gene simultaneously except three *bla*_*CTX–M–*64_-bearing plasmids from strains 400, 1966, and 3354 (Supplementary Table S1). The IncI1-type replicon can be detected in four out of 23 *bla*_*CTX–M–*199_-carrying plasmids and one out of 40 *bla*_*CTX–M–*64_-carrying plasmids. Remarkably, it can also be detected in all four *bla*_*CTX–M–*123_-carrying plasmids and all five *bla*_*CTX–M–*153_-carrying plasmids. These plasmids were closely related to a *bla*_*CTX–M–*123_-bearing IncI1 plasmid pHNAH4-1 (GenBank accession no. NC_024955.2) which was obtained from an *E. coli* isolated from the stool sample of a chicken in Anhui province of China (Liu et al., 2015). Seven *bla*_*CTX–M–*199_-bearing and four *bla*_*CTX–M–*64_-bearing plasmids with ca. 110 kb in size belonged to IncFIB type (Supplementary Table S1 and Figure 2C). Interestingly, the sequences of pM-199-1028 and pM-64-1028, both harbored in the same strain 1028, were almost identical, differing only by two nucleotide substitutions that distinguish *bla*_*CTX–M–*199_ from *bla*_*CTX–M–*64_ gene (Figure 2C). Similar plasmids can also be found in *E. coli* isolated from anal swab and feed samples from a breeding goose farm in China, such as pL100-3 (GenBank accession no. NZ_CP034747.1) and pL41-1-3 (GenBank accession no. NZ_CP034729.1), two IncFIB plasmids carrying *bla*_*CTX–M–*199_, which were identified in Jiangsu province recently (Liu et al., 2019). The IncHI2-type replicon was only detected in *bla*_*CTX–M–*64_-bearing plasmids in this study (Supplementary Figure S2).

### Genetic Context of *bla*_*CTX–M–*1/9/1_ Genes

**FIGURE 3:**
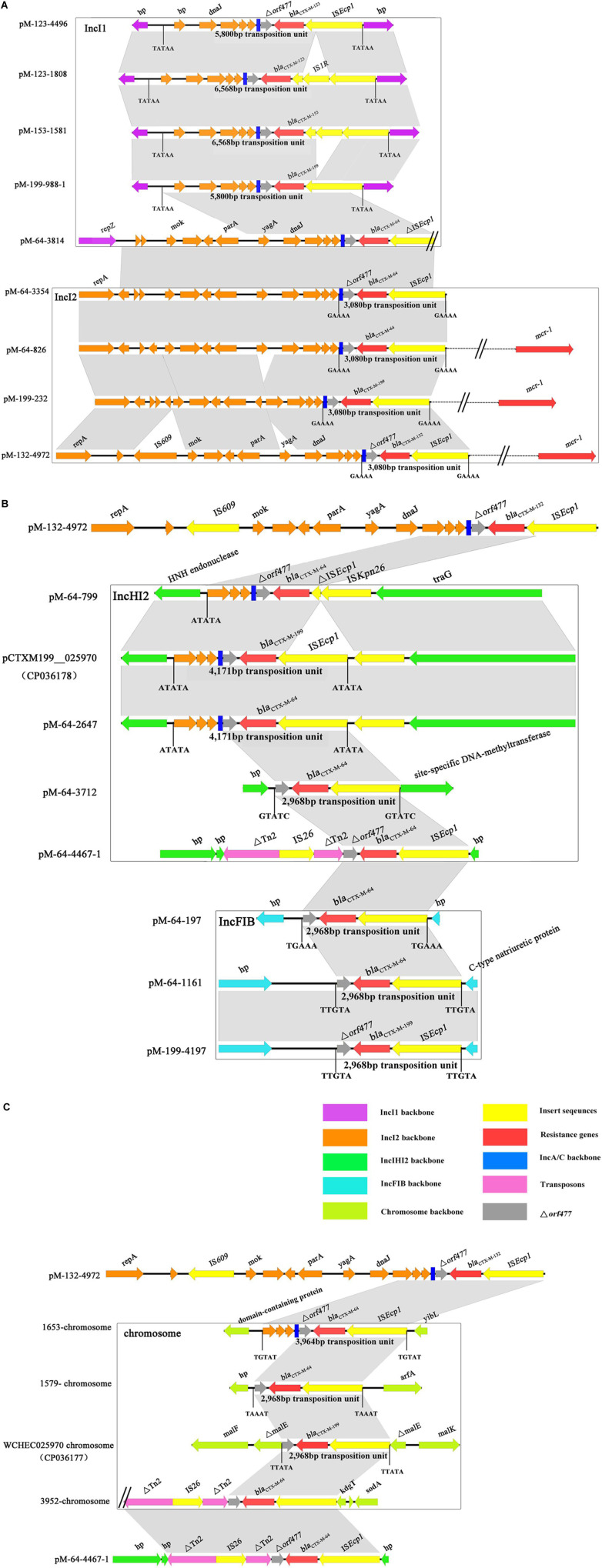
Alignment of the genetic environment surrounding *bla*_*CTX–M–*1/9/1_ genes located on different Inc types of plasmids **(A,B)** and chromosomes **(C)**. The genes of different function or different origins were labeled with different colors. The position and sizes of the transposition units were indicated. Only the representative sequences of plasmids and chromosomes were compared. The sequences of the remaining isolates shared the same or similar genetic structure of the *bla*_*CTX–M–*1_,_9_,_1_ genes with that of the representative sequences and were listed in Supplementary Table S3.

Based on a comparison of the genetic environment surrounding *bla*_*CTX–M*–1/9/1_ genes, transposition units flanked by different direct repeats were determined in all samples (Figure 3). IS*Ecp1* was present in all transposition units; however, their sizes varied. Apart from the 3,080- and 5,800-bp IS*Ecp1* transposition units located on IncI2 and IncI1 plasmids, respectively, a 2,968 bp transposition unit was identified in the plasmids of IncFIB and IncHI2 and chromosome. For the IncHI2 plasmid and chromosome, more than one type of transposition units can be found. These results suggested that *bla*_*CTX–M*–1/9/1_ genes can be carried by an IS*Ecp1* mobile genetic element in various sizes of transposition units and disseminated among different types of plasmids and chromosomes.

## Discussion

The intestinal tract is an important reservoir and source for transmission of many antibiotic resistance genes, including *bla* genes, and intestinal colonization is associated with infections caused by ESBL-producing organisms (Donskey, 2004; Carlet, 2012; Karanika et al., 2016). This study revealed a low carriage rate of *bla*_*CTX–M–*199_, which was located on three types of plasmids—IncI2, IncFIB, and IncI1—in healthy populations of a Chinese city. However, the *bla*_*CTX–M–*199_ gene can be located on IncHI2-type plasmid by searching the NCBI database. One example was the pCTXM199_025970 (GenBank accession no. CP036178.1) (Figure 2D), which was harbored in an *E. coli* isolated from Sichuan province. A large number of CTX-M-199 producers recovered from clinical specimens have been found in different hospitals, including two sporadic *E. coli* isolates from Zhejiang and Sichuan provinces (Li et al., 2018) (GenBank accession no. CP036178.1) and several *E. coli*, *K. aerogenes*, and *K. quasivariicola* isolates from multiple samples of the same patient in another hospital of Zhejiang province (GenBank accession nos. VLPD01000017.1, VLOS01000013.1, VLOY01000014.1, and VLPG01000031.1). CTX-M-199 has also been found in *E. coli* from goose, *Gallus*, and chicken in three different provinces (Jiangsu, Jilin, and Zhejiang) (Liu et al., 2019) (GenBank accession nos. CP034747.1, CP034729.1, RRZS01000003.1, and RDCW01000100.1) and *K. pneumoniae* from hospital sewage in Sichuan province (GenBank accession nos. NQVM01000026 and NQVL01000026). The majority of these isolates harbored the IncI2 plasmid, rare harbored the IncFIB and IncHI2 plasmids, and none harbored the IncI1 plasmid. Among the 22 IncI2-type plasmids identified in this study, 19 carried the *mcr-1* gene (except three plasmids from strains 400, 1966, and 3354). Twenty-two *E. coli* isolates (including 12 CTX-M-199 producers, eight CTX-M-64 producers, and two CTX-M-132 producers) were positive for *mcr-1* gene, 19 of which were located on IncI2 plasmids (except three *mcr-1* from strains 197, 1079, and 3814). The coexistence of two resistance genes—*mcr-1* and *bla*_*CTX–M*–1/9/1_—in the same IncI2 plasmid may increase the risk of co-selection of this plasmid type and may contribute to the predominance of IncI2 plasmid among isolates of various origins.

The phylogenetic analysis showed that no significant association was found between a specific clone and the production of CTX-M-1/9/1. However, two remarkable dominant STs of *E. coli*, ST48, which was frequently identified in NDM and ESBL producers (Smet et al., 2010; Liu et al., 2019), and ST746, which was the most prevalent type of CTX-M-123 producers from chicken farms of China (Liu et al., 2015), were observed. ST48 and its derivative can be detected in multiple CTX-M-1/9/1 producers (five CTX-M-64, three CTX-M-199, one CTX-M-123, and one CTX-M-153), two of which co-produced NDM. Unlike isolates from chicken, three of four ST746 *E. coli* produced CTX-M-64 (Supplementary Table S1). The presence of each subtype of *bla*_*CTX–M–*1/9/1_ gene in *E. coli* from different clades in the phylogenetic tree based on SNPs (Figure 1) indicated that *E. coli* of diverse genetic backgrounds contributed to the dissemination of *bla*_*CTX–M–*1/9/1_ genes, although few clonal spreads seemed to have occurred.

The *bla*_*CTX–M–*199_ gene may be generated *via* two possible routes. One possibility was that CTX-M-199 was derived directly from CTX-M-64 by two substitutions of A^109^T and S^130^T. The *E. coli* 1028 in this study appeared to support this hypothesis. In this isolate, we found the coexistence of two 112,177-bp plasmids (pM-199-1028 and pM-64-1028 carrying *bla*_*CTX–M–*199_ and *bla*_*CTX–M–*64_ genes, respectively), which differed by only two bases. However, no S^130^T mutation was found in CTX-M variants which were resistant to β-lactamase inhibitors by *in vitro* selection (Ripoll et al., 2011). Consistent with this, the serial passage of *E. coli* strains, each carrying pM-64-1028 and three other *bla*_*CTX–M–*64_-carrying plasmids with different Inc types at growing concentrations of cefoperazone/sulbactam, also failed to generate S^130^T mutation (data not shown). Thus, the *bla*_*CTX–M–*199_ gene was more likely to be formed by the recombination between *bla*_*CTX–M–*1–like_ and *bla*_*CTX–M–*14–like_ carrying natural S^130^T mutation. The generated inhibitor-resistant *bla*_*CTX–M–*1/9/1_ hybrid gene further disseminated among different types of plasmids *via* IS*Ecp1*-mediated transposition. As one of the most important elements associated with the mobilization and dissemination of *bla*_*CTX–M*_ genes, transposition units mediated by IS*Ecp1* varied (Zhao and Hu, 2013). Two IS*Ecp1*-mediated transposition units, with 3,080 and 5,800 bp, respectively, identified in this study can also be found in the previous study (Liu et al., 2015). Our current data were unable to depict the evolution of *bla*_*CTX–M–*1/9/1_ hybrid gene. However, from the analysis of the genetic backgrounds of these types of genes carrying different gene cassettes and plasmids, we confirmed that IS*Ecp1* was the most common element that mediated the transmission of these types of genes. Allele change between different IS*Ecp1*-mediated *bla*_*CTX–Ms*_ mobile elements might facilitate the formation of hybrid *bla*_*CTX–M*_ genes.

One limitation of this study is that the risk factors for CTX-M-199 carriage are not assessed, as information on occupation, diet, behavioral characteristics, or travel from the participants were not available.

## Conclusion

This study reported the intestinal colonization of healthy populations of China by CTX-M-1/9/1-producing *E. coli* with a fecal carriage rate of 1.36%. The *bla*_*CTX–M–*1/9/1_ gene, carried by the mobile element IS*Ecp1*, was located on four major types of plasmids and disseminated among *E. coli* with diverse genetic backgrounds. Our findings provide insights into the dissemination and further development of appropriate control measures for this inhibitor-resistant β-lactamase.

## Data Availability Statement

The data presented in the study are deposited in GenBank and accession numbers can be found in the Supplementary Material.

## Ethics Statement

This study was approved by the Ethics Committee of The Second Affiliated Hospital of Zhejiang University School of Medicine, and written informed consent was given by participants.

## Author Contributions

JCa and SC conceived and designed the work. JCh and YJ provided samples and collected enterococcal isolates. JCh performed the whole-genome sequencing and sequence assembly. JCa, JCh, and SC analyzed the data and interpreted the results. JCh and JCa drafted the manuscript. SC and RZ improved the English. All authors revised the manuscript and approved the final version.

## Conflict of Interest

The authors declare that the research was conducted in the absence of any commercial or financial relationships that could be construed as a potential conflict of interest.

## Footnotes

1 https://www.fda.gov/drugs/development-resources/tigecycline-injection-products

2 https://cge.cbs.dtu.dk/services/PlasmidFinder/

3 https://itol.embl.de/
