# Supplementary material for: Fecal Carriage and Genetic Characterization of CTX-M-1/9/1-Producing Escherichia coli From Healthy Humans in Hangzhou, China
Source: Front Microbiol. 2021 Feb 16;12:616687. doi: 10.3389/fmicb.2021.616687 (PMC7921147; doi:10.3389/fmicb.2021.616687)
Supplement: Supplementary file 1 [file Data_Sheet_1.docx]

**Supplementary Table S1.** Characterization of 74 CTX-M-1/9/1-producing Enterobacterales isolated from 68 stool samples of 5,000 healthy human in Hangzhou, China

| Isolate | Species | ST type ^a^ | Participant | | *bla*_CTX-M-1/9/1_-containing fragment | | | Acquired antibiotic resistance genes |
| --- | --- | --- | --- | --- | --- | --- | --- | --- |
|  |  |  | Gender | Age | Location | Size | Transferability^b^ |  |
| 232 | *E. coli* | ST162 | Male | 80 | IncI2 plasmid | 65,345bp, circular | **+** | ***bla_CTX-M-199_, mcr-1*** |
| 427 | *E. coli* | ST155 | Male | 43 | IncFIB plasmid | 111,358bp, circular | **+** | ***bla_CTX-M-199_****, bla_TEM-1_, bla_OXA-10_, qnrS1, tet(A), floR, cmlA1, ARR-2, dfrA14, aadA1* |
| 522 | *E. coli* | ST7153 | Male | 30 | IncI2 plasmid | 65,845bp, circular | **+** | ***bla_CTX-M-199_, mcr-1****, bla_TEM-1_, fosA3, oqxAB, tet(A), floR, mph(A), dfrA12, sul1, sul2, rmtB, aadA2, aph(6)-Id, aph(3')-IIa, aph(3'')-Ib* |
| 879 | *E. coli* | ST616 | Female | 46 | IncI2 plasmid | 65,845bp, circular | **+** | ***bla_CTX-M-199_, mcr-1****, bla_TEM-1_, bla_OXA-10_, qnrS1, tet(A), cmlA1, lnu(F), ARR-2, dfrA14, dfrA17, sul1, sul2, sul3, aac(3)-IVa, aac(3)-IIe, aadA1, aadA5, aph(4)-Ia, aph(6)-Id, aph(3')-Ia, aph(3'')-Ib* |
| 988-1 | *E. coli* | ST181 | Female | 46 | IncI1 plasmid | 103,844bp, linear | **+** | ***bla_CTX-M-199_****, bla_TEM-176_, fosA3, qnrS1, tet(A), floR, mph(A), dfrA14, aph(3')-Ia* |
| 988-2 | *E. coli* | ST2973 |  |  | IncI1 plasmid | 102,472bp, linear | **+** | ***bla_CTX-M-199_****, bla_CTX-M-55_, fosA3, tet(A), floR, mph(A), dfrA17, sul2, aadA5, aph(6)-Id, aph(3'')-Ib* |
| 988-3 | *E. coli* | ST226 |  |  | IncI1 plasmid | 102,472bp, linear | **+** | ***bla_CTX-M-199_****, bla_TEM-1_, fosA3, tet(A), floR, mph(A), lnu(F), dfrA12, sul1, sul2, sul3, aac(3)-IId, aadA2, aph(3')-Ia* |
| 1002 | *E. coli* | ST224 | Male | 72 | IncI1 plasmid | 95,860bp, linear | **+** | ***bla_CTX-M-199_****, fosA3, tet(A), floR, mph(A), sul2, aac(3)-IId, aph(6)-Id, aph(3'')-Ib* |
| 1028 | *E. coli* | ST48 | Male | 36 | IncFIB plasmid | 112,177bp, circular | **+** | ***bla_CTX-M-199_, bla_CTX-M-64_****, bla_NDM-5_, bla_OXA-1_, qnrS2, oqxAB, aac(6')-Ib-cr, catB3, ARR-3* |
| T1028-S | *E. coli* transconjugant | ST10 | / | / | IncFIB plasmid | 112,177bp, circular | **+** | ***bla_CTX-M-199_*** |
| T1028-A | *E. coli* transconjugant | ST10 | / | / | IncFIB plasmid | 112,177bp, circular | **+** | ***bla_CTX-M-64_*** |
| 1066 | *E. coli* | ST48* | Male | 35 | IncI2 plasmid | 65,846bp, circular | **+** | ***bla_CTX-M-199_, mcr-1****, bla_CTX-M-14_, bla_TEM-135_, fosA3, qnrS1, tet(A), floR, dfrA17, sul2, aac(3)-IVa, aph(4)-Ia, aph(3')-Ia* |
| 1230 | *E. coli* | ST48* | Male | 34 | IncI2 plasmid | 64,932bp, circular | **+** | ***bla_CTX-M-199_, mcr-1****, bla_CTX-M-14_, bla_TEM-176_, fosA3, qnrS1, tet(A), floR, dfrA14, sul2, aac(3)-IVa, aph(4)-Ia, aph(3')-Ia* |
| 1247 | *E. coli* | ST46 | Female | 53 | IncI2 plasmid | 65,373bp, linear | **+** | ***bla_CTX-M-199_, mcr-1****, bla_TEM-1_, tet(A), tet(B), floR, catA1, dfrA17, sul2, aph(6)-Id, aph(3'')-Ib* |
| 1269 | *E. coli* | ST1249 | Male | 40 | IncFIB plasmid | 112,942bp, circular | **+** | ***bla_CTX-M-199_*** |
| 1771-1 | *E. coli* | ST58 | Male | 37 | IncFIB plasmid | 114,139bp, circular | **+** | ***bla_CTX-M-199_****, bla_TEM-176_, qnrS1, tet(A), floR, dfrA14, aph(3')-Ia* |
| 1771-2 | *E. coli* | ST746 |  |  | IncFIB plasmid | 112,469bp, circular | **+** | ***bla_CTX-M-199_****, tet(B), aph(6)-Id, aph(3'')-Ib* |
| 2137 | *E. coli* | ST2732 | Male | 30 | IncI2 plasmid | 66,753bp, linear | **+** | ***bla_CTX-M-199_, mcr-1****, bla_CTX-M-55_, Δbla_TEM-1_, fosA3, oqxAB, tet(A), floR, mph(A), dfrA1, sul1, sul2, aadA1, aph(6)-Id, aph(3')-IIa, aph(3'')-Ib* |
| 2199 | *E. coli* | NF | Male | 54 | IncI2 plasmid | 65,567bp, circular | **+** | ***bla_CTX-M-199_, mcr-1****, bla_CTX-M-55_, Δbla_TEM-1_* |
| 2203 | *E. coli* | ST1431 | Male | 50 | IncI2 plasmid | 65,845bp, circular | **+** | ***bla_CTX-M-199_, mcr-1****, bla_TEM-1_, bla_OXA-10_, qnrS1, tet(A), floR, cmlA1, lnu(F), ARR-2, dfrA17, sul3, aac(3)-IVa, aadA2, aph(4)-Ia, aph(6)-Id, aph(3')-Ia, aph(3'')-Ib* |
| 2296 | *E. coli* | NF | Male | 39 | IncI2 plasmid | 62,938bp, circular | **+** | ***bla_CTX-M-199_, mcr-1****, bla_CTX-M-55_, Δbla_TEM-1_, oqxAB, tet(A), floR, sul2, aph(6)-Id, aph(3')-IIa, aph(3'')-Ib* |
| 2539 | *E. coli* | ST10 | Female | 25 | IncFIB plasmid | 112,176bp, circular | **+** | ***bla_CTX-M-199_,*** *bla_TEM-1_, bla_OXA-10_, qnrS1, tet(A), floR, cmlA1, mph(A), ARR-2, dfrA14, sul2, sul3, aadA1, aadA2, aph(6)-Id, aph(3'')-Ib* |
| 2654 | *E. coli* | NF | Male | 31 | IncI2 plasmid | 64,935bp, circular | **+** | ***bla_CTX-M-199_, mcr-1****, bla_CTX-M-55_, Δbla_TEM-1_, oqxAB, tet(A), floR, sul2, aph(6)-Id, aph(3')-IIa, aph(3'')-Ib* |
| 2694 | *E. coli* | ST69 | Female | 70 | IncI2 plasmid | 65,861bp, circular | **+** | ***bla_CTX-M-199_, mcr-1****, bla_CTX-M-14_, bla_TEM-1_, tet(A), tet(M), floR, cmlA1, lnu(F), dfrA12, sul2, sul3, aac(3)-IVa, aac(3)-IId, aadA1, aadA2, aph(4)-Ia, aph(3')-Ia* |
| 4197 | *E. coli* | ST1638 | Female | 56 | IncFIB plasmid | 112,469bp, circular | **+** | ***bla_CTX-M-199_****, bla_TEM-176_, qnrS1, tet(A), floR, erm(B), dfrA14, aadA22, aph(3')-Ia* |
| 86 | *E. coli* | ST1266 | Male | 37 | chromosome | 161,680bp, linear | **-** | ***bla_CTX-M-64_****, tet(A), tet(B), floR, cmlA1, catA1, dfrA24, sul1, sul2, aadA1, aph(6)-Id, aph(3'')-Ib, ant(2'')-Ia* |
| 197 | *E. coli* | ST448 | Male | 77 | IncFIB plasmid | 111,112bp, circular | **+** | ***bla_CTX-M-64_****, mcr-1, bla_CTX-M-55_, Δbla_TEM-1_, fosA3, qnrS1, oqxAB, tet(A), tet(M), floR, mph(A), sul2, aac(3)-IVa, aph(4)-Ia, aph(6)-Id, aph(3')-IIa, aph(3'')-Ib* |
| 400 | *E. coli* | ST457 | Female | 46 | IncI2 plasmid | 15,200bp, linear | **+** | ***bla_CTX-M-64_****, bla_TEM-1_, fosA3, oqxAB, tet(A), floR, mph(A), dfrA12, sul1, sul2, rmtB, aadA2, aph(6)-Id, aph(3')-IIa, aph(3'')-Ib* |
| 799 | *E. coli* | ST2913 | Female | 36 | IncHI2 plasmid | 184,610bp, linear | **+** | ***bla_CTX-M-64_****, bla_TEM-1_, oqxAB, tet(A), floR, mph(A), ARR-3, dfrA12, dfrA27, sul1, sul2, aac(3)-IId, aadA2, aadA16, aph(6)-Id, aph(3'')-Ib* |
| 826 | *E. coli* | ST617 | Male | 81 | IncI2 plasmid | 66,334bp, linear | **+** | ***bla_CTX-M-64_, mcr-1****, bla_TEM-1_, fosA3, oqxAB, tet(A), floR, mph(A), dfrA12, sul1, sul2, rmtB, aadA2, aph(6)-Id, aph(3')-IIa, aph(3'')-Ib* |
| 1079 | *E. coli* | ST224 | Male | 54 | IncHI2 plasmid | 109,981bp, linear | **+** | ***bla_CTX-M-64_****, mcr-1, bla_CTX-M-55_, bla_TEM-1_, bla_OXA-10_, fosA3, qnrS1, oqxAB, tet(A), floR, cmlA1, mph(A), ARR-2, dfrA14, dfrA27, sul1, rmtB, aac(3)-IId, aadA1, aadA16, aph(3')-IIa* |
| 1153 | *E. coli* | ST457 | Male | 63 | chromosome | 6,113bp, linear | **-** | ***bla_CTX-M-64_****, bla_TEM-1_, tet(A), floR, cmlA1, mph(A), sul2, sul3, dfrA12, dfrA14, aac(3)-IIa, aadA1, aadA2, aph(6)-Id, aph(3')-Ia, aph(3'')-Ib* |
| 1161 | *E. coli* | NF | Female | 62 | IncFIB plasmid | 110,879bp, circular | **+** | ***bla_CTX-M-64_****, bla_TEM-135_, tet(A), qnrS1, floR* |
| 1324 | *E. coli* | ST2973 | Male | 55 | IncI2 plasmid | 59,693bp, linear | **+** | ***bla_CTX-M-64_, mcr-1****, bla_TEM-1_, oqxAB, tet(A), tet(M), floR, cmlA1, mph(A), erm(B), dfrA12, sul1, sul2, sul3, rmtB, aac(3)-IVa, aadA1, aadA2, aph(6)-Id, aph(3')-IIa, aph(3'')-Ib* |
| 1579 | *E. coli* | ST746 | Male | 34 | chromosome | 228,217bp, linear | **-** | ***bla_CTX-M-64_****, bla_TEM-1_, fosA3, oqxAB, tet(A), floR, mph(A), dfrA12, sul1, sul2, rmtB, aadA2, aph(6)-Id, aph(3')-IIa, aph(3'')-Ib* |
| 1653 | *E. coli* | ST349 | Female | 36 | chromosome | 209,640bp, linear | **-** | ***bla_CTX-M-64_****, bla_TEM-1_, tet(A), mph(A), erm(B), dfrA12, sul1, sul2, aadA2, aac(3)-IIa, aph(6)-Id, aph(3'')-Ib* |
| 1953 | *E. coli* | ST746 | Female | 25 | IncHI2 plasmid | 186,193bp, linear | **+** | ***bla_CTX-M-64_****, bla_TEM-1_, qnrS1, oqxAB, tet(A), floR, dfrA17* |
| 1966 | *E. coli* | ST69 | Male | 63 | IncI2 plasmid | 66,577bp, linear | **+** | ***bla_CTX-M-64_****, dfrA14* |
| 2322 | *E. coli* | ST1193 | Male | 28 | chromosome | 11,232bp, linear | **-** | ***bla_CTX-M-64_****, bla_TEM-1_, tet(A), mph(A), dfrA17, sul1, sul2, aac(3)-IId, aadA5, aph(6)-Id, aph(3'')-Ib* |
| 2340-1 | *E. coli* | NF | Male | 31 | IncFIB plasmid | 110,879bp, circular | **+** | ***bla_CTX-M-64_****, bla_NDM-5_, bla_TEM-135_, qnrS1, tet(A), floR, dfrA14* |
| 2340-2 | *E. coli* | ST167 |  |  | IncHI2 plasmid | 132,851bp, linear | **+** | ***bla_CTX-M-64_****, bla_TEM-1_, fosA3, oqxAB, tet(A), floR, dfrA17, sul1, sul2, rmtB, aac(6')-Ib, aadA2, aadA5* |
| 2358 | *E. coli* | ST48 | Male | 32 | chromosome | 88,391bp, linear | **-** | ***bla_CTX-M-64_****, bla_NDM-5_, bla_TEM-1_, qnrB6, aac(6')-Ib-cr, tet(A), floR, mph(A), ARR-3, dfrA17, dfrA27, sul1, sul2, aac(3)-IId, aadA5, aadA16, aph(6)-Id, aph(3'')-Ib* |
| 2360 | *E. coli* | ST359 | Male | 54 | IncI2 plasmid | 64,950bp, linear | **+** | ***bla_CTX-M-64_, mcr-1****, bla_TEM-1_, qnrS1, tet(A), tet(M), floR, cmlA1, dfrA12, sul2, sul3, aac(3)-VIa, aadA1, aadA2, aph(3')-Ia* |
| 2475-1 | *E. coli* | ST48 | Male | 75 | chromosome | 88,390bp, linear | **-** | ***bla_CTX-M-64_****, bla_TEM-135_, tet(A), floR, dfrA17, sul2, aadA5, aph(6)-Id, aph(3'')-Ib* |
| 2475-2 | *E. coli* | ST2973 |  |  | IncI2 plasmid | 63,714bp, linear | **+** | ***bla_CTX-M-64_, mcr-1****, bla_TEM-1_, fosA3, tet(A), floR, mph(A), erm(B), dfrA12, sul1, sul2, rmtB, aadA2, aph(6)-Id, aph(3')-IIa, aph(3'')-Ib* |
| 2480 | *E. coli* | ST617 | Male | 34 | IncHI2 plasmid | 181,376bp, linear | **+** | ***bla_CTX-M-64_****, bla_TEM-1_, aac(6')Ib-cr, tet(A), floR, mph(A), ARR-3, sul1, sul2, dfrA27, rmtB, aac(3)-IId, aadA16, aph(6)-Id, aph(3')-IIa, aph(3'')-Ib* |
| 2550 | *Klebsiella aerogenes* | ST127 | Male | 38 | IncHI2 plasmid | 164,505bp, linear | **+** | ***bla_CTX-M-64_****, bla_TEM-1_, bla_LAP-2_, fosA3, qnrS1, tet(A), floR, dfrA14, sul1, sul2, sul3, rmtB, aac(6')-Ib, aadA2, aph(3')-Ia* |
| 2647 | *E. coli* | ST746 | Male | 34 | IncHI2 plasmid | 186,194bp, linear | **+** | ***bla_CTX-M-64_****, bla_TEM-135_, qnrS1, tet(A), floR, dfrA12, dfrA17, sul3, aadA2, ΔaadA5* |
| 2998 | *E. coli* | ST48 | Female | 38 | chromosome | 18,758bp, linear | **-** | ***bla_CTX-M-64_****, bla_TEM-1_, fosA4, qnrS1, aac(6')Ib-cr, tet(A), floR, mph(A), ARR-3, sul1, dfrA5, dfrA14, dfrA17, dfrA27, aadA5, aadA16* |
| 3199 | *E. coli* | ST2178 | Male | 41 | IncI2 plasmid | 65,233bp, linear | **+** | ***bla_CTX-M-64_, mcr-1****, bla_CTX-M-14_, mph(A), dfrA17, aadA5, aac(3)-IId* |
| 3354 | *E. coli* | ST349 | Female | 57 | IncI2 plasmid | 60,600bp, circular | **+** | ***bla_CTX-M-64_****, bla_CTX-M-15_, qnrS1* |
| 3463 | *E. coli* | ST48 | Female | 50 | chromosome | 88,390bp, linear | **-** | ***bla_CTX-M-64_****, bla_TEM-135_, tet(A), floR, dfrA17, sul2, aadA5, aph(6)-Id, aph(3'')-Ib* |
| 3524 | *E. coli* | NF | Male | 29 | chromosome | 228,478bp, linear | **-** | ***bla_CTX-M-64_****, bla_CTX-M-55_, bla_TEM-1_, qnrS1, oqxAB, tet(A), tet(M), floR, mph(A), dfrA12, sul2, aadA2, aph(6)-Id, aph(3')-IIa, aph(3'')-Ib* |
| 3653 | *E. coli* | ST6823 | Male | 41 | chromosome | 233,470bp, linear | **-** | ***bla_CTX-M-64_****, bla_TEM-1_, bla_OXA-1_, qnrS2, oqxAB, aac(6')Ib-cr, tet(A), floR, catB3, mph(A), ARR-3, dfrA27, sul1, sul2, aac(3)-IIa, aadA16, aph(6)-Id, aph(3')-Ia, aph(3'')-Ib* |
| 3712 | *E. coli* | NF | Male | 38 | IncHI2 plasmid | 106,562bp, linear | **+** | ***bla_CTX-M-64_****, bla_NDM-5_, bla_OXA-10_, qnrS1, qnrS2, tet(A), floR, cmlA1, ARR-2, dfrA14, aadA1* |
| 3814 | *E. coli* | ST1011 | Male | 63 | IncI1 plasmid | 103,879bp, linear | **+** | ***bla_CTX-M-64_****, mcr-1, bla_TEM-1_, bla_OXA-10_, fosA3, oqxAB, tet(A), floR, cmlA1, mph(A), ARR-2, dfrA14, sul1, sul2, aadA1, aac(3)-IId, aph(3')-IIa* |
| 3940 | *E. coli* | NF | Female | 52 | chromosome | 28,030bp, linear | **-** | ***bla_CTX-M-64_****, bla_TEM-135_, bla_OXA-10_, tet(A), floR, cmlA1, ARR-2, dfrA14, sul2, aadA1, aph(3')-IIa* |
| 3952 | *E. coli* | ST167 | Female | 39 | chromosome | 67,189bp, linear | **-** | ***bla_CTX-M-64_****, bla_TEM-176_, qnrS1, tet(A), floR, dfrA14, aph(3')-Ia* |
| 4079 | *E. coli* | ST48 | Female | 30 | IncHI2 plasmid | 187,725bp, linear | **+** | ***bla_CTX-M-64_****, bla_OXA-10_, qnrS1, tet(A), floR, cmlA1, mph(A), ARR-3, dfrA14, dfrA27, sul1, sul2, aadA1, aadA16, aph(3')-IIa* |
| 4289 | *E. coli* | ST6823 | Male | 40 | chromosome | 277,209bp, linear | **-** | ***bla_CTX-M-64_****, tet(A), floR, mph(A), ARR-3, dfrA27, sul1, sul2, aac(3)-IIa, aadA16, aph(6)-Id, aph(3')-Ia, aph(3'')-Ib* |
| 4467-1 | *E. coli* | ST218 | Male | 58 | IncHI2 plasmid | 152,804bp, linear | **+** | ***bla_CTX-M-64_****, bla_TEM-1_, fosA3, floR, sul1, rmtB, aadA2, aph(6)-Id, aph(3')-Ia, aph(3'')-Ib* |
| 4467-2 | *Enterobacter cloacae* | ST116 |  |  | IncHI2 plasmid | 132,851bp, linear | **+** | ***bla_CTX-M-64_****, bla_TEM-1_, bla_ACT-7_, fosA3, floR, sul1, rmtB, aadA2, aph(6)-Id, aph(3'')-Ib* |
| 4512 | *E. coli* | ST533 | Female | 31 | IncHI2 plasmid | 134,873bp, linear | **+** | ***bla_CTX-M-64_****, bla_TEM-1_, bla_OXA-1_, fosA3, tet(A), floR, catB3, ARR-3, dfrA5, dfrA17, sul1, sul2, rmtB, aac(3)-IId, aac(6')-Ib, aadA2, aadA5, aph(3')-Ia* |
| 4851 | *E. coli* | ST1629 | Female | 52 | IncHI2 plasmid | 187,883bp, linear | **+** | ***bla_CTX-M-64_****, bla_CTX-M-14_, bla_TEM-1_, bla_OXA-1_, aac(6')-Ib-cr, tet(A), tet(B), floR, catA1, catB3, lnu(G), ARR-3, sul1, sul2, aac(3)-IId, aph(6)-Id, aph(3')-Ia, aph(3'')-Ib* |
| 1808 | *E. coli* | ST457 | Female | 49 | IncI1 plasmid | 114,325bp, linear | **+** | ***bla_CTX-M-123_****, bla_CTX-M-65_, bla_TEM-1_, tet(M), floR, cmlA1, lnu(F), dfrA12, sul2, sul3, aac(3)-IVa, aac(3)-IId, aadA1, aph(4)-Ia* |
| 1986 | *E. coli* | ST48 | Female | 61 | chromosome | 19,847bp, linear | **-** | ***bla_CTX-M-123_****, bla_CTX-M-65_, Δbla_TEM-1_, bla_OXA-10_, qnrS1, tet(A), floR, cmlA1, ARR-2, dfrA14, aadA1* |
| 2700 | *E. coli* | ST1771 | Male | 75 | IncI1 plasmid | 106,117bp, linear | **+** | ***bla_CTX-M-123_****, bla_TEM-1_, fosA3, oqxAB, tet(A), floR, mph(A), dfrA12, sul1, sul2, rmtB, aadA2, aph(6)-Id, aph(3')-IIa, aph(3'')-Ib* |
| 3438 | *E. coli* | NF | Male | 32 | IncI1 plasmid | 102,084bp, linear | **+** | ***bla_CTX-M-123_****, bla_CTX-M-55_, bla_TEM-1_, fosA3, qnrS1, tet(A), floR, catA1, mph(A), lnu(F), ARR-2, dfrA12, dfrA14, sul1, sul2, sul3, rmtB, aac(3)-IId, aadA2, aadA22, aph(6)-Id, aph(3')-Ia, aph(3'')-Ib* |
| 4496 | *E. coli* | ST156 | Male | 43 | IncI1 plasmid | 102,640bp, linear | **+** | ***bla_CTX-M-123_****, bla_TEM-1_, fosA3, tet(A), tet(B), catA1, catB3, mph(A), dfrA1, sul1, aac(6')-Ib* |
| 1581 | *E. coli* | ST4943 | Male | 69 | IncI1 plasmid | 88,929bp, linear | **+** | ***bla_CTX-M-153_****, bla_CTX-M-55_, bla_CTX-M-65_, bla_TEM-1_, qnrS1, oqxAB, tet(A), floR, mph(A), lnu(F), dfrA17, sul1, sul2, sul3, aac(3)-IVa, aac(3)-IId, aadA5, aadA22, aph(4)-Ia, aph(3')-Ia* |
| 1585 | *E. coli* | ST48 | Male | 52 | IncI1 plasmid | 86,877bp, linear | **+** | ***bla_CTX-M-153_****, bla_CTX-M-65_, bla_TEM-1_, bla_OXA-10_, fosA3, tet(A), floR, cmlA1, catA1, mph(A), ARR-2, dfrA14, dfrA17, sul1, sul2, rmtB, aadA1, aadA5, aph(3')-IIa* |
| 3772 | *E. coli* | ST1139 | Female | 26 | IncI1 plasmid | 103,248bp, linear | **+** | ***bla_CTX-M-153_****, bla_NDM-1_, fosA3, qnrS1, mph(A), dfrA14* |
| 4465 | *E. coli* | ST3944 | Female | 53 | IncI1 plasmid | 61,067bp, linear | **+** | ***bla_CTX-M-153_****, bla_NDM-5_, fosA3, oqxAB, tet(A), tet(B), floR, mph(A), ARR-3, dfrA27, sul1, sul2, aadA16, aph(6)-Id, aph(3')-IIa, aph(3'')-Ib* |
| 4510 | *E. coli* | ST8128 | Male | 58 | IncI1 plasmid | 91,172bp, linear | **+** | ***bla_CTX-M-153_****, fosA3, oqxAB, tet(A), floR, mph(A), sul2, aac(3)-IId, aph(6)-Id, aph(3')-IIa, aph(3'')-Ib* |
| 3832 | *E. coli* | ST457 | Male | 56 | IncI2 plasmid | 64,185bp, linear | **+** | ***bla_CTX-M-132_, mcr-1****, bla_TEM-1_, fosA3, oqxAB, tet(A), floR, mph(A), erm(B), dfrA12, sul1, sul2, rmtB, aadA2, aph(6)-Id, aph(3')-IIa, aph(3'')-Ib* |
| 4972 | *E. coli* | ST156 | Female | 57 | IncI2 plasmid | 65,579bp, circular | **+** | ***bla_CTX-M-132_, mcr-1****, bla_CTX-M-14_, bla_TEM-1_, fosA3, oqxAB, tet(A), floR, catA1, mph(A), dfrA12, dfrA17, sul1, sul2, rmtB, aadA2, aadA5, aac(3)-IVa, aph(4)-Ia, aph(6)-Id, aph(3')-IIa, aph(3'')-Ib* |

^a^ ST48* indicated that ST48 was the nearest ST type; NF, sequence type of the *E. coli* isolate was not found.

^b^ + indicated that the conjugation experiment was successful; – indicated that the conjugation experiment failed.

**Supplementary Table S2.** Antimicrobial susceptibility results of 74 *bla*_CTX-M-1/9/1_-positive isolates

| Antimicrobial Agent ^a^ | *bla*_CTX-M-199_-positive *E. coli* (n=23) ^b^ | | | | Other *bla*_CTX-M-1/9/1_-positive isolates (n=51) | | | | Total (n=74) | | | |
| --- | --- | --- | --- | --- | --- | --- | --- | --- | --- | --- | --- | --- |
|  | MIC Range  (mg/liter) | MIC_50_  (mg/liter) | MIC_90_  (mg/liter) | Resistance rate | MIC Range  (mg/liter) | MIC_50_  (mg/liter) | MIC_90_  (mg/liter) | Resistance rate | MIC Range  (mg/liter) | MIC_50_  (mg/liter) | MIC_90_  (mg/liter) | Resistance rate |
| imipenem | ≤0.25-32 | ≤0.25 | 0.5 | 4.3% | ≤0.25-16 | ≤0.25 | 0.5 | 9.8% | ≤0.25-32 | ≤0.25 | 0.5 | 8.1% |
| meropenem | ≤0.25-64 | ≤0.25 | ≤0.25 | 4.3% | ≤0.25-64 | ≤0.25 | ≤0.25 | 9.8% | ≤0.25-64 | ≤0.25 | ≤0.25 | 8.1% |
| ertapenem | ≤0.25-64 | ≤0.25 | ≤0.25 | 4.3% | ≤0.25-64 | ≤0.25 | ≤0.25 | 9.8% | ≤0.25-64 | ≤0.25 | ≤0.25 | 8.1% |
| cefotaxime | 64->128 | >128 | >128 | 100% | 64->128 | >128 | >128 | 100% | 64->128 | >128 | >128 | 100% |
| cefotaxime/clavulanate | ≤1/0.5-64/32 | ≤1/0.5 | 2/1 | 4.3% | ≤1/0.5-64/32 | ≤1/0.5 | 2/1 | 9.8% | ≤1/0.5-64/32 | ≤1/0.5 | 2/1 | 8.1% |
| ceftazidime | 4->128 | 8 | 16 | 26.1% | 16->128 | 64 | >128 | 100% | 4->128 | 32 | >128 | 77.0% |
| ceftazidime/clavulanate | ≤1/0.5-64/32 | ≤1/0.5 | 2/1 | 4.3% | ≤1/0.5-64/32 | ≤1/0.5 | 2/1 | 9.8% | ≤1/0.5-64/32 | ≤1/0.5 | 2/1 | 8.1% |
| piperacillin/tazobactam | 32/4-256/4 | 64/4 | 128/4 | 30.4% | ≤4/4-256/4 | ≤4/4 | 64/4 | 9.8% | ≤4/4-256/4 | 16/4 | 128/4 | 16.2% |
| cefoperazone/sulbactam | 32/16->128/64 | 32/16 | 64/32 | 47.8% | ≤4/2->128/64 | 16/8 | 32/16 | 9.8% | ≤4/2->128/64 | 32/16 | 64/32 | 21.6% |
| ceftazidime/avibactam | ≤0.5/4->64/4 | ≤0.5/4 | 1/4 | 4.3% | ≤0.5/4->64/4 | ≤0.5/4 | 4/4 | 9.8% | ≤0.5/4->64/4 | ≤0.5/4 | 2/4 | 8.1% |
| cefmetazole | ≤0.5-64 | ≤0.5 | 4 | 4.3% | ≤0.5->128 | 2 | 64 | 27.5% | ≤0.5->128 | 2 | 64 | 20.3% |
| aztreonam | ≤1->128 | ≤1 | 64 | 26.1% | 16->128 | 128 | >128 | 100% | ≤1->128 | 64 | >128 | 77.0% |
| ciprofloxacin | ≤0.25-32 | 1 | 32 | 56.5% | ≤0.25->32 | 16 | >32 | 70.6% | ≤0.25->32 | 4 | 32 | 66.2% |
| gentamicin | ≤1->64 | ≤1 | 64 | 21.7% | ≤1->64 | 64 | >64 | 60.8% | ≤1->64 | 8 | >64 | 48.6% |
| tigecycline | ≤0.5 | ≤0.5 | ≤0.5 | 0% | ≤0.5 | ≤0.5 | ≤0.5 | 0% | ≤0.5 | ≤0.5 | ≤0.5 | 0% |
| colistin | 0.5-8 | 4 | 8 | 52.2% | ≤0.25-8 | 0.5 | 4 | 19.6% | ≤0.25-8 | 0.5 | 4 | 29.7% |

^a^ For piperacillin/tazobactam and ceftazidime/avibactam, the tazobactam and avibactam were tested at a fixed concentration of 4 mg/liter. For cefotaxime/clavulanate, ceftazidime/clavulanate, and cefoperazone/sulbactam, the combinations were tested with concentrations of 2:1 ratio (antibiotic: inhibitor).

^b^ *E. coli* 1028 which produced both CTX-M-199 and CTX-M-64 was assigned to the *bla*_CTX-M-199_-positive group.

**Supplementary Table S3.** Sequences of the *bla*_CTX-M-1,9,1_-carrying plasmids and chromosomal fragments and their GenBank accession numbers

| GenBank accession number | The submitted *bla*_CTX-M-1,9,1_-containing sequence | Other *bla*_CTX-M-1,9,1_-containing sequences with high homology to submitted sequences |
| --- | --- | --- |
| MT773664 | pM-199-232 | pM-199-522, pM-199-879, pM-199-2203, pM-199-1066, pM-199-1247, pM-199-2137, pM-199-2199, pM-199-2296, pM-199-2654, pM-199-1230, pM-199-2694 |
| MT773665 | pM-123-4496 | pM-123-2700, pM-123-3438 |
| MT773666 | pM-123-1808 | None |
| MT773667 | pM-153-1581 | pM-153-1585, pM-153-3772, pM-153-4465, pM-153-4510 |
| MT773668 | pM-199-988-1 | pM-199-988-2, pM-199-988-3, pM-199-1002 |
| MT773669 | pM-64-3814 | None |
| MT773670 | pM-64-3354 | pM-64-400, pM-64-1966 |
| MT773671 | pM-132-4972 | pM-132-3832 |
| MT773672 | pM-64-197 | None |
| MT773673 | pM-64-1161 | pM-64-2340-1 |
| MT773674 | pM-199-4197 | pM-199-427, pM-199-1028, pM-199-2539, pM-199-1771-1, pM-199-1771-2, pM-199-1269 |
| MT773675 | pM-64-826 | pM-64-1324, pM-64-2360, pM-64-2475-2, pM-64-3199 |
| MT773676 | pM-64-799 | pM-64-1079, pM-64-2480 |
| MT773677 | pM-64-2647 | pM-64-1953, pM-64-4079, pM-64-4851 |
| MT773678 | pM-64-3712 | None |
| MT773679 | pM-64-4467-1 | pM-64-2340-2, pM-64-2550, pM-64-4467-2, pM-64-4512 |
| MT773680 | 1653-chromosome | None |
| MT773681 | 1579-chromosome | 86-chromosome, 1153-chromosome, 2322-chromosome, 2358-chromosome, 2475-1-chromosome, 2998-chromosome, 3463-chromosome, 3524-chromosome, 3653-chromosome, 3940-chromosome, 4289-chromosome, 1986-chromosome |
| MT773682 | 3952-chromosome | None |

**Supplementary Figure S1.** The faecal carriage of *bla*_CTX-M-1/9/1_ and *bla*_CTX-M-199_ genes in different age groups of healthy adults


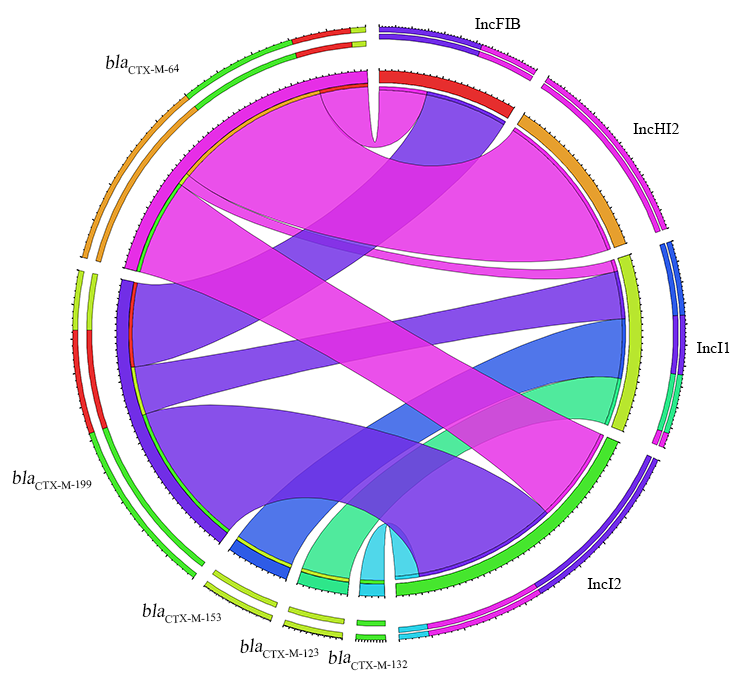


**Supplementary Figure S2.** A chord diagram showing the distribution of *bla*_CTX-M-1/9/1_ genes carried by four Inc-types of plasmids and the distribution of Inc-types of plasmids carrying different *bla*_CTX-M-1/9/1_ genes. Different *bla*_CTX-M-1/9/1_ genes and different Inc-types of plasmids were displayed by various colors along the left-hand side and the right-hand side, respectively. The length of the circular line represented the relative number of *bla*_CTX-M-1/9/1_ gene or *bla*_CTX-M-1/9/1_-carrying plasmid.
